# Mobile-based interventions for common mental disorders in youth: a systematic evaluation of pediatric health apps

**DOI:** 10.1186/s13034-021-00401-6

**Published:** 2021-09-13

**Authors:** Matthias Domhardt, Eva-Maria Messner, Anna-Sophia Eder, Sophie Engler, Lasse B. Sander, Harald Baumeister, Yannik Terhorst

**Affiliations:** 1 Department of Clinical Psychology and Psychotherapy, Institute of Psychology and Education, Ulm University, Lise-Meitner-Str. 16, 89081 Ulm, Germany; 2 Department of Rehabilitation Psychology and Psychotherapy, Institute of Psychology, Albert-Ludwigs-University Freiburg, Engelberger Str. 41, 79106 Freiburg im Breisgau, Germany; 3 Department of Research Methods, Institute of Psychology and Education, Ulm University, Albert-Einstein-Allee 47, 89069 Ulm, Germany

**Keywords:** mHealth, Internet, Smartphone, Psychotherapy, Children, Adolescent, Anxiety, Depression, Posttraumatic stress disorder

## Abstract

**Background:**

The access to empirically-supported treatments for common mental disorders in children and adolescents is often limited. Mental health apps might extend service supplies, as they are deemed to be cost-efficient, scalable and appealing for youth. However, little is known about the quality of available apps. Therefore, we aimed to systematically evaluate current mobile-based interventions for pediatric anxiety, depression and posttraumatic stress disorder (PTSD).

**Methods:**

Systematic searches were conducted in *Google Play Store* and *Apple App Store* to identify relevant apps. To be eligible for inclusion, apps needed to be: (1) designed to target either anxiety, depression or PTSD in youth (0–18 years); (2) developed for children, adolescents or caregivers; (3) provided in English or German; (4) operative after download. The quality of eligible apps was assessed with two standardized rating systems (i.e., *Mobile App Rating Scale (MARS)* and *ENLIGHT*) independently by two reviewers.

**Results:**

Overall, the searches revealed 3806 apps, with 15 mental health apps (0.39%) fulfilling our inclusion criteria. The mean overall scores suggested a moderate app quality (MARS: M = 3.59, SD = 0.50; ENLIGHT: M = 3.22, SD = 0.73). Moreover, only one app was evaluated in an RCT. The correlation of both rating scales was high (*r* = .936; p < .001), whereas no significant correlations were found between rating scales and user ratings (*p* > .05).

**Conclusions:**

Our results point to a rather poor overall app quality, and indicate an absence of scientific-driven development and lack of methodologically sound evaluation of apps. Thus, future high-quality research is required, both in terms of theoretically informed intervention development and assessment of mental health apps in RCTs. Furthermore, institutionalized best-practices that provide central information on different aspects of apps (e.g., effectiveness, safety, and data security) for patients, caregivers, stakeholders and mental health professionals are urgently needed.

**Supplementary Information:**

The online version contains supplementary material available at 10.1186/s13034-021-00401-6.

## Background

Mental disorders frequently originate in early periods of life [1], and are associated with significant burden and personal suffering [2]. Anxiety, depression and posttraumatic stress disorder are prevalent in children and adolescents, with increasing incidence rates worldwide [3–5]. Without effective and timely treatments, these common mental disorders tend to continue into adulthood, with augmented risks to develop further mental disorders and other detrimental outcomes in the long-term [e.g., 6].

Empirically-supported treatment options for common mental disorders in youth (0–18 years) embrace psychotherapy conveyed face-to-face [e.g., 7] and pharmacotherapy [e.g., 8]. In particular, cognitive-behavioral therapy (CBT) has comprehensively documented its effectiveness and acceptability in the treatment of anxiety disorders [9], depression [10, 11] and PTSD in children and adolescents [12]. However, the availability and uptake rates of conventional evidence-based psychotherapies remain low [13], due to different individual and structural barriers, like shortage of mental healthcare supplies, long-waiting times, limitations in mobility, and high treatment costs or fear of stigmatization [14, 15]. Thus, innovative and scalable solutions to lessen this treatment gap are urgently needed; and these novel interventions have to prove their empirical support in terms of various aspects of evidence-based medicine/psychotherapy, such as efficacy and effectiveness, quality or intervention safety.

Internet- and mobile-based interventions (IMIs) hold the potential to lessen existing gaps in mental healthcare [13, 16], as they feature several advantages that might help to overcome some of these obstructions to treatment. First, structural barriers might be reduced, as IMIs can be implemented irrespective of the restraints of space and time [17, 18]. Second, IMIs are deemed to be cost-efficient, as they might be scalable for large numbers of patients [19, 20]. Third, IMIs might also diminish some individual barriers, because these interventions can reduce stigma threat by preserving anonymity, and allow patients to integrate them flexibly into their daily lives at their own pace and individual progress [13, 21]. Furthermore, the digital delivery mode of these novel interventions might be especially appealing for youth, who are frequently familiar and acquainted with the Internet and its applications on mobile devices from an early age [13, 22].

The evidence-base for the efficacy of internet-based psychotherapeutic interventions for some common mental disorders in children and adolescents is documented by several meta-analyses so far [13]. The strongest empirical support can be found for anxiety disorders in youth with effect sizes ranging from 0.30 to 0.77 (standardized mean differences (SMD); 95% CI − 0.53 to 1.45) derived from five meta-analyses [23–27]. Furthermore, the efficacy of internet-based interventions for depression in youth is documented by four meta-analyses [23, 24, 27, 28], with effect sizes in the range from 0.16 to 0.76 (SMD; 95% CI − 0.12 to 1.12). Aside from this meta-analytical evidence, there is are also preliminary indications that internet-based interventions might be acceptable [29] and cost-effective [30], and hold the potential to extend service supplies within a framework of stepped mental healthcare [29, 31].

Given the efficacy of internet-based psychotherapeutic interventions and the worldwide dispersal of smartphones, mobile health applications (MHA) become more and more appealing as a way to complement or deliver psychotherapeutic support [19]. Recent meta-analyses indicate that also app-based psychotherapeutic interventions are efficacious [32, 33]: For instance, for depressive symptoms (SMD = 0.28, 95% CI 0.21 to 0.36), generalized anxiety symptoms (SMD = 0.30, 95% CI 0.20 to 0.40) [32] or smoking behavior (SMD = 0.39, 95% CI 0.21 to 0.57) [33]. However, studies provide mainly evidence for adults. Of the k = 66 [32] and k = 19 [33] included studies in the meta-analyses on the efficacy of app-based psychotherapeutic interventions, only one focused on youth [34].

The limited evidence on mobile health apps for children and adolescents seems like the more important, as there is already a vast (evidence-free) commercial app market accessible in routine care: In an international cooperation study, the analysis of N = 1.299 commercially available MHA yielded, that 94.8% were not evidence-based [35]. Besides major limitations towards the evidence-base in the app markets, systematic evaluations about the quality of available MHA highlight a high heterogeneity and weak to moderate quality of MHA, especially regarding the engagement of users and information quality [16, 35–38]. Moreover, privacy and security features of MHA available in the app markets have been identified as crucial issues for patients’ safety [39, 40].

Hence, it is of utmost importance to evaluate the apps available in the commercial app market to highlight the few high-quality, secure and scientifically evaluated MHA to inform and guide users as well as healthcare professionals. Standardized and reliable instruments like the Mobile Application Rating Scale (MARS) [35, 41, 42] or the ENLIGHT instrument [43] that offer an assessment for quality, privacy features, and evidence-base enable such evaluations. Although previous reviews have systematically evaluated health apps for depression [16] and PTSD [36] in adults, to our knowledge, no such study was conducted relating to mobile-based interventions targeted for common mental disorders in children and adolescents to date. Therefore, this study aimed to systematically evaluate the quality of existing apps that were explicitly designated to address symptoms of anxiety, depression and PTSD in youth, by deploying two established psychometric app rating scales [41–43]. Specifically, we aimed to:

1. systematically evaluate the quality of eligible apps in the European *Google Play Store* and *Apple App Store* regarding user engagement, functionality, aesthetics, and information content;
2. assess privacy and security features;
3. identify theoretical foundations of apps and their respective intervention components;
4. assess the concordance of user ratings and expert ratings.

## Methods

### Search strategy and inclusion criteria

A systematic search for MHA targeting anxiety, depression and posttraumatic stress disorder (PTSD) in children and adolescents was conducted by using an automatic search engine (webcrawler) that was developed within the mobile health app database project (MHAD; http://mhad.science/ [16, 36]. The webcrawler is a program that searches the app stores for given search terms and automatically extracts the provided information (e.g., app name, app description, user ratings, etc.) from the stores. The functionality and validity of the program has been proven in previous studies [44–46]. For further technical details please see [47].

In the present study, three sets of search terms were used to identify MHA anxiety, depression and PTSD in youth (age range: 0–18 years; Additional file 1). The search terms were derived by a team of psychologists (ASE, MD, YT), among them a licensed psychotherapist for children and adolescents (MD) and an expert for MHA quality (YT; [16, 35, 36]). The systematic searches (both, in English and German) were conducted from April 24th to April 26th, 2019.

After the app identification, a two-step inclusion process was conducted. First all identified apps were screened based on the app title and description regarding the following inclusion criteria: (a) the app was developed to address at least one of the targeted mental disorders, (b) the app was focusing on children and/or their caregivers, (c) the app was available in a language spoken by the authors (German or English), (d) the app was available for download. Second, all remaining apps were downloaded and tested against the criteria a-c again and e) the app was operative. All eligible MHA were included in the current analysis.

### Assessment

The Mobile Application Rating Scale (MARS German version [42]) and the ENLIGHT are two standardized and reliable scales to assess the contents and quality of MHA. In the present analysis app characteristics (e.g., app name, price, user-rating) were assessed using the classification site of the standardized MARS [41, 42]. In addition, the classification site was used to assess methods (e.g., mindfulness exercises) and the therapeutic background. Based on previous categorizations [16, 48] therapeutic background was rated as cognitive behavioral therapy (CBT), psychodynamic psychotherapy, behavioral therapy, systemic therapy, third-wave CBT, humanistic therapy, integrative therapy, other, not applicable (N.A.; e.g., mainly psychoeducational content), or mixed (e.g., elements from multiple therapeutic backgrounds and theoretical orientations).

The MARS offers several items to assess privacy and security features (e.g., password protection) on a descriptive level. All included apps were rated by these items. In addition, the ENLIGHT instrument contains four checklists (i.e., privacy, security, credibility, evidence-based score), providing further in-depth assessment domains. For privacy and security, explicit requirements were stated, which are either rated as fulfilled or unfilled. Credibility and evidence-base is rated from very poor to excellent. For further information see [41].

### Quality rating

The MARS [41, 42] was used for the quality assessment. With the MARS, MHA quality is rated on a 5-point scale ranging from 1 “inadequate” to 5 “excellent”. A total of 19 items on four sub-dimensions were included: (A) engagement (5 items: fun, interest, individual adaptability, interactivity, target group), (B) functionality (4 items: performance, usability, navigation, gestural design), (C) aesthetics (3 items: layout, graphics, visual appeal), and (D) information quality (7 items: accuracy of app description, goals, quality of information, the quantity of information, quality of visual information, credibility, evidence base). The psychometric quality of the MARS is excellent [35, 41, 42]. To assess the evidence base of the included apps, each reviewer conducted a search using Google and Google Scholar (search terms: app name, efficacy, effectiveness, observation study, study, evaluation, usability) and searched on the website of the app and app provider for information on conducted studies. Based on the search results the evidence items was rated as NA (“The app has not been trialled/tested”), 1 (“The evidence suggests the app does not work”), 2 (“App has been trialled (e.g., acceptability, usability, satisfaction ratings) and has partially positive outcomes in studies that are not randomised controlled trials (RCTs), or there is little or no contradictory evidence”), 3 (“App has been trialled (e.g., acceptability, usability, satisfaction ratings) and has positive outcomes in studies that are not RCTs, and there is no contradictory evidence“), 4 (“App has been trialled and outcome tested in 1–2 RCTs indicating positive results”), or 5 (“App has been trialled and outcome tested in > 3 high quality RCTs indicating positive results”) [41]. In addition to the four objective quality scales, the MARS contains two subjective subscales: (E) subjective quality (4 items: recommendation, frequency of use, willingness to pay, overall star-rating) and (F) perceived impact (6 items: awareness, knowledge, attitudes, intention to change, help-seeking, behavioural change).

In addition to the MARS, the ENLIGHT instrument was applied, since it provides information on additional dimensions (e.g., therapeutic alliance). ENLIGHT’s items are rated on a 5-point scale ranging from 1 “very poor” to 5 “very good”. In total ENLIGHT covers 28 items on seven sub-dimensions: (a) usability (3 items), (b) visual design (3 items), (c) user engagement (5 items), (d) content (4 items), (e) therapeutic persuasiveness (7 items), (f) therapeutic alliance (3 items), and (g) general subjective evaluation (3 items). The internal consistency of ENLIGHT is excellent [43].

### Rater training

The quality rating was conducted by two independent reviewers (ASE, SE). All reviewers completed online training for the MARS before the quality assessment (https://www.youtube.com/watch?v=5vwMiCWC0Sc; last updated on September 6, 2021). For the ENLIGHT instrument, no free online training is available. Instead, reviewers were trained in using the ENLIGHT by an researcher with prior experience and expertise in app quality assessment (YT; [16, 35, 36]. After the instruction, several test apps were rated by both reviewers (ASE, SE) and ratings were discussed between the reviewers and instructor (YT) to complete the training phase. The excellent inter-rater reliability between the trained reviewers for the MARS (ICC = 0.87, 95% CI 0.84 to 0.90) and the inter-rater reliability for the ENLIGHT (ICC = 0.75, 95% CI 0.65 to 0.83) highlight that the training was successful.

### Data analyses

MHA characteristics, therapeutic backgrounds, methods, and privacy and security features were analyzed descriptively. As a measure of agreement between the trained reviewers, intra-class correlation (ICC) was calculated for the dimensional quality assessment [49]. An ICC above 0.75 was defined as a satisfactory agreement between the reviewers [50]. The ratings of both reviewers were pooled. After pooling, means and standard deviations were calculated for all quality dimensions of the MARS and ENLIGHT. The associations between user ratings (star-rating in the stores: 5-point rating from 1 star to 5 stars) and experts’ quality ratings with the MARS and ENLIGHT were investigated by correlation analysis. For correlation analysis, an alpha level of 5% was defined. Holm correction for multiple testing was applied [51]. Missing values were excluded list-wise.

## Results

### Search and app characteristics

**Fig. 1:**
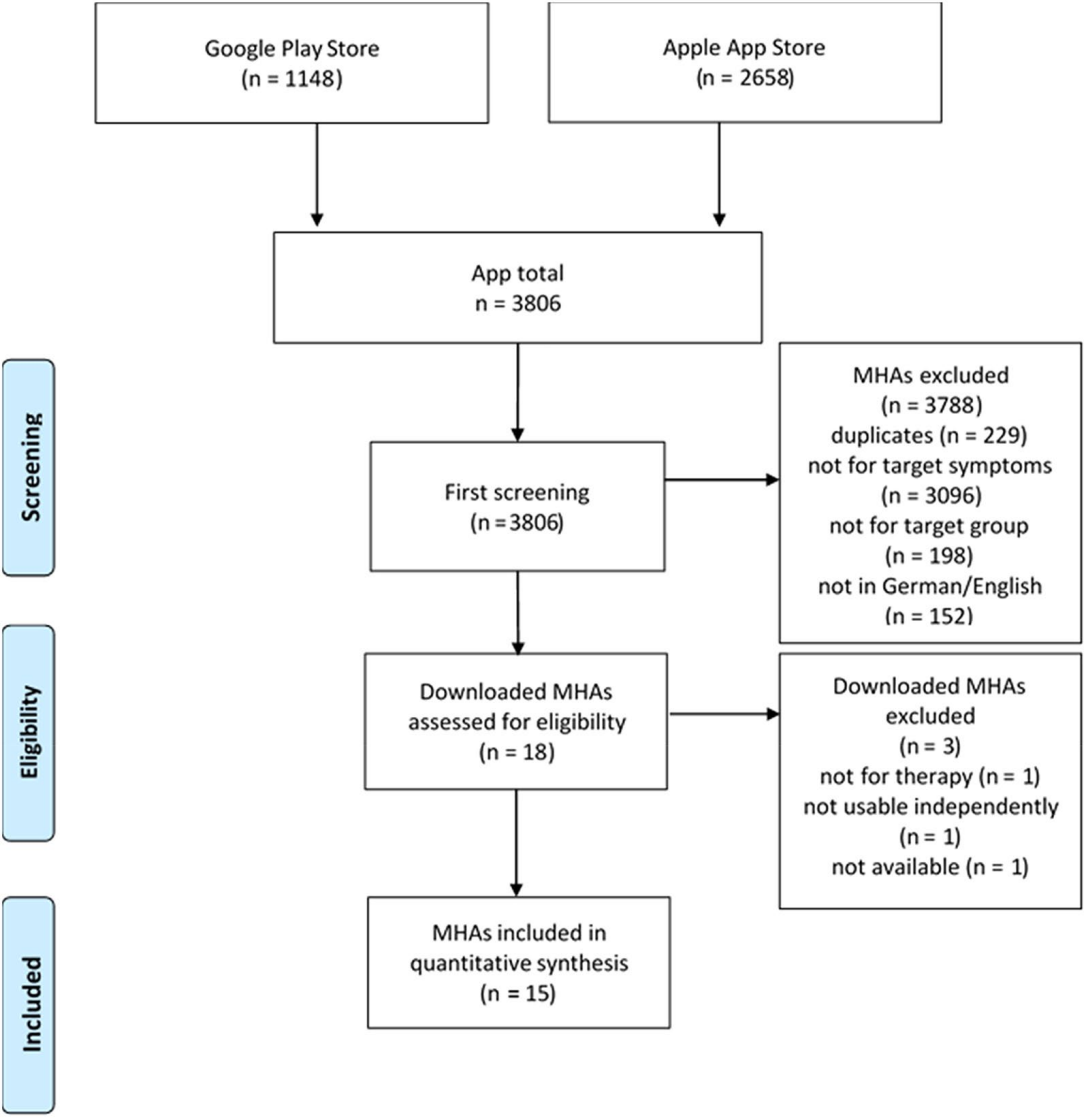
Flow-chart of the in- and exclusion process

In total, 3806 MHA were identified (Apple App Store = 2658, Play Store = 1148). After the eligibility check, 15 MHA were included. All MHA were developed for Apple devices and n = 7 MHA were also available on Android. The inclusion process is summarized in Fig. 1.

Most MHA (n = 9, 60%) were from the category *Health and Fitness*, followed by n = 6 (40%) from the category *Medicine*. *Lifestyle* (n = 3), *Education* (n = 2) and *social networks* (n = 1) were represented categories as well. Of the 15 included MHA, two targeted depression, four anxiety, three PTSD. The remaining six targeted mixed symptoms of anxiety, depression and PTSD. Most MHA were freely available (n = 9, 60%). Prices of the six fee-based MHA (40%) ranged from 1.09 EUR to 10.99 EUR. On average, users rated the included MHA moderate to good (M = 3.6; SD = 1.04); three MHA were not rated by users. MHA characteristics are summarized in Table 1.

**Table 1:** App characteristics

Name	Version	Store	User rating	Number of ratings	Category	Price
Anxiety Coach	1.3.6	Apple	2.5	8	Health & fitness	5,50 EUR
BoosterBuddy	1.6	AppleGoogle Play	4.4	27	Health & fitness, lifestyle	Free
Braineka Panic Attacks	2	Apple	NA	0	Medicine	3,49 EUR
ClearFear	1.3	AppleGoogle Play	4.3	12	Healt & fitness	Free
Depression Test and Training	3.5.10	Apple	3.6	5	Medicine,Health &Fitness	In-App purchases
EMDR Therapy	0	Apple	4.0	1	Medicine	10,99 EUR
eReading: Sam	0	Apple	1.0	1	Education	2,29 EUR
FearShrinker	1.3	Apple	NA	0	Medicine	6,99 EUR
iChill	2.1	AppleGoogle Play	4.1	13	Health & fitness	Free
iPrevail	3.6.8	AppleGoogle Play	3.3	36	Health & fitnesssocial networks	In-App purchases
Kids’ Grief	1.5	Apple	NA	0	Lifestyle	1,99 EUR
Parent-Guide	1.0.0	AppleGoogle Play	5.0	1	Education medicine	Free
Talkspace OnlineTherapy	8.53.03	AppleGoogle Play	3.6	11	Health & fitnessmedicine	In-App purchases
Teen Counseling	7.1	AppleGoogle Play	4.1	82	Health &fitness, lifestyle	In-App purchases
The Emotion Diary	2.3	Apple	3.3	3	Health & fitness, lifestyle	In-App purchases

**Fig. 2:**
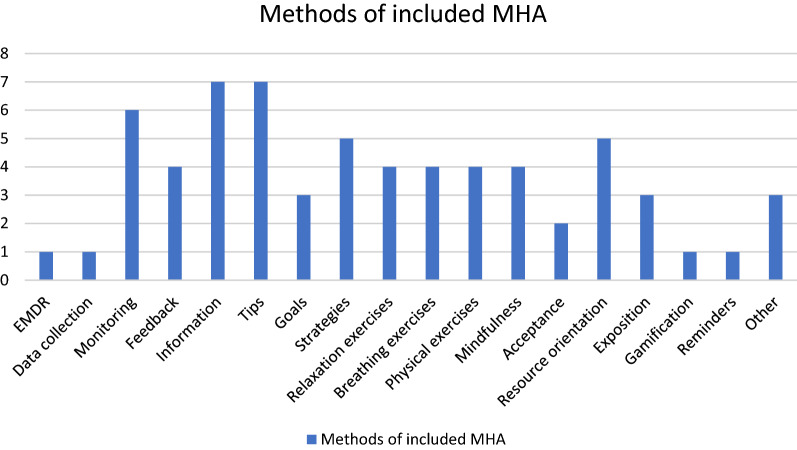
Methods and functions of included MHA

The investigation of methods provided by the included MHA yielded 18 different features. Most frequently, MHA provided information (n = 7) and offered psycho-educational advice (n = 7). A total of n = 6 MHA (40%) included a monitoring function. MHA also included therapeutic functions. For an aggregated overview of all functions, see Fig. 2.

In four apps the used functions and exercises were taken from multiple therapeutic backgrounds. A clear CBT focus was observed in two MHA, while at least some CBT elements (including third-wave CBT) were present in six of the MHA. Interestingly, a total of three apps incorporated the contact to a therapist or coach, which may lead to different therapeutic backgrounds and applied techniques depending on the assigned therapist or coach. An MHA-specific summary of the provided functions as well as the therapeutic background of the MHA is provided in Table 2.

**Table 2:** Content summary

Name	Keywords	Methods and functions	Therapeutic background
Anxiety coach	Anxiety disorder, cognitive behavioral therapy, self-confidence	Monitoring, feedback, information, tips, goals, exposition	CBT
BoosterBuddy	Mental health, coping, establishing positive behavior	Monitoring, feedback, information, tips, goals, strategies, relaxation, mindfulness, acceptance, resource orientation, reminders	Mixed (with elements of CBT, behavioral therapy, third-wave CBT)
Braineka Panic Attacks	Panic attacks, anxiety disorder, exposition	Relaxation, breathing exercises, exposition	Mixed (with elements of CBT, third-wave CBT, behavioral therapy)
ClearFear	Anxiety disorder, resilience, relaxation	Tips, relaxation, breathing exercises, physical exercises, resource orientation	CBT
Depression test and training	Depression, diagnosis, cognitive training	Data collection, monitoring, feedback, information, strategies, gamification	Other (Neuropsychology)
EMDR therapy	support trauma therapy, EMDR	EMDR	Other (EMDR)
eReading: Sam	narrative, life with PTSD, trauma	Information, strategies, breathing exercises, physical exercises	N.A. (Psychoeducation)
FearShrinker	Anxiety disorder, strategies, coping	Monitoring, feedback, information, tips, goals, strategies, relaxation, breathing exercises, physical exercises, exposition	Mixed (with elements of CBT, behavioral therapy)
iChill	Resilience-model, self help, stress, trauma	Monitoring, physical exercises, mindfulness, acceptance, resource orientation	Other (Community Resilience Model)
iPrevail	Community support, anonymous chat, peer group	Tips	Various depending on therapist/coach
Kids’ grief	Advice for parents, coping with grief, support	Information, tips, resource orientation	N.A. (Psychoeducation)
Parent-guide	Traumatic event, strategies, security	Information, tips, strategies, mindfulness, other	N.A. (Psychoeducation)
Talkspace online therapy	“Match” with an online therapist	Other	Various depending on therapist/coach
Teen counseling	“Match” with an online therapist	Other	Various depending on therapist/coach
The emotion diary	Positive psychology, diary, tracking	Monitoring, mindfulness, resource orientation	Mixed (with elements of positive psychology and third-wave CBT)

### Privacy, security and evidence

Of all 15 included MHA, n = 6 (40%) offered password protection, n = 4 (27%) required a log-in, n = 7 (47%) had a visible privacy policy, and n = 7 (47%) had an imprint. Consent to data collection was required in an active form by n = 5 (33%) MHA and in a passive form by n = 8 (53%), n = 1 informed about conflicts of interest/financial background. An emergency function was provided by n = 2 (13%) MHA. The ENLIGHT checklist evaluation of privacy, security, credibility, and evidence-base is summarized in Table 3.

In addition to the ENLIGHT checklist, evidence-base was assessed by MARS with the item “Has the app been trialled/tested?”. The assessment revealed, that only a single MHA (“iChill”) has been evaluated and outcome tested in an RCT.

**Table 3:** Checklist summary ENLIGHT

App	Privacy	Security	Credibility	Evidence-based score
FearShrinker	X	X	NA	Very poor
ClearFear	X	X	Poor	Very poor
Anxiety Coach	X	X	Fair	Very poor
Braineka Panic Attacks	X	X	Fair	Very poor
Depression Test und Training	X	X	Fair	Very poor
Kids’ Grief	X	X	NA	Very poor
Parent-Guide	X	X	Good	Very poor
eReading: Sam	X	X	NA	Very poor
EMDR Therapy	X	X	NA	Very poor
BoosterBuddy	X	X	Fair	Very poor
Teen Counseling	X	X	Fair	Very poor
Talkspace Online Therapy	X	X	Good	Very poor
iChill	X	X	Fair	Fair
iPrevail	X	X	Poor	Very poor
The Emotion Diary	X	X	NA	Very poor

X represents, that requirements by the ENLIGHT instrument were not met. Credibility was rated as NA, if credibility could not be accounted for (see ENLIGHT [43])

### Quality rating

#### MARS

The overall average quality of MHA was M = 3.59 (SD = 0.50). Quality ratings of the four sub-dimensions resulted in: engagement M = 3.34 (SD = 0.76), functionality M = 3.81 (SD = 0.49), aesthetics M = 3.78 (SD = 0.63), information quality M = 3.42 (SD = 0.68). The subjective quality was M = 3.13 (SD = 0.62) and the perceived impact M = 2.80 (SD = 0.75). Detailed information can be obtained from Table 4.

**Table 4:** MARS rating

App	Engagement	Functionality	Aesthetics	Information quality	Overall	Subjective quality	Perceived impact
FearShrinker	4.4	4.50	4.33	3.33	4.14	3.75	3.17
ClearFear	4.4	4.13	4.33	3.42	4.07	3.63	3.42
Anxiety Coach	3.3	3.5	3.33	3.70	3.46	3.13	3.33
Braineka Panic Attacks	2.9	3.88	4.17	3.58	3.63	2.75	2.08
Depression Test und Training	3.5	3.88	4.50	3.71	3.90	2.88	2.33
Kids’ Grief	2.8	3.25	2.83	3.50	3.10	3.00	3.58
Parent-Guide	2.8	4.25	4.00	3.60	3.66	3.38	2.75
eReading: Sam	3.5	3.50	3.50	2.33	3.21	3.25	3.92
EMDR Therapy	2.9	2.50	3.17	3.00	2.89	2.00	1.00
BoosterBuddy	4.5	4.13	4.50	4.13	4.31	4.00	3.42
Teen Counseling	3.9	4.25	4.50	2.92	3.89	3.88	3.00
Talkspace OnlineTherapy	3.6	4.00	4.00	5.00	4.15	3.75	2.83
iChill	1.9	3.63	3.33	3.67	3.13	2.88	2.42
iPrevail	3.6	3.88	3.50	3.30	3.57	2.13	2.75
The Emotion Diary	2.1	3.88	2.67	2.17	2.70	2.50	2.00
	3.34 (SD = 0.76)	3.81 (SD = 0.49)	3.78 (SD = 0.63)	3.42 (SD=0.68)	3.59 (SD = 0.50)	3.13 (SD = 0.62)	2.80 (SD = 0.75)

#### ENLIGHT

The overall quality was M = 3.22 (SD = 0.73). Quality ratings of the sub-dimensions yielded: usability M = 3.84 (SD = 0.39), visual design M = 3.72 (SD = 0.55), user engagement M = 3.27 (SD = 0.80), content M = 3.34 (SD = 0.72), therapeutic persuasiveness M = 2.34 (SD = 0.78), therapeutic alliance M = 2.56 (SD = 1.08), general subjective evaluation M = 3.34 (SD = 0.83). For detailed information see Table 1.

**Table 5:** ENLIGHT rating

App	Usability	Design	User engagement	Content	Therapeutic persuasiveness	Therapeutic alliance	Subjective evaluation
FearShrinker	4.83	4.33	4.20	3.75	3.21	3.33	3.83
ClearFear	4.00	4.83	4.10	4.00	3.29	3.00	4.33
Anxiety Coach	3.50	3.33	3.10	3.75	3.21	1.67	3.33
Braineka Panic Attacks	4.17	3.83	2.50	3.50	2.14	2.83	3.00
Depression Test und Training	4.00	4.17	3.70	3.38	2.70	2.17	3.50
Kids’ Grief	3.67	2.83	2.50	3.75	2.00	2.17	3.17
Parent-Guide	4.00	3.83	2.20	3.88	2.14	2.67	3.33
eReading: Sam	3.50	3.67	2.80	3.50	1.64	3.50	3.67
EMDR Therapy	3.50	3.33	2.40	2.00	1.50	1.00	2.17
BoosterBuddy	4.00	4.33	4.70	4.38	4.00	4.50	4.33
Teen Counseling	4.00	4.00	3.80	3.75	NA^1^	NA^1^	4.83
Talkspace Online Therapy	4.00	3.67	3.80	3.25	NA^1^	NA^1^	3.67
iChill	3.33	3.33	2.50	2.75	1.93	1.67	2.17
iPrevail	3.83	3.33	3.90	2.50	2.17	3.83	2.83
The Emotion Diary	3.33	3.00	2.80	2.00	1.67	1.00	2.00
	3.84 (SD = 0.39)	3.72 (SD = 0.55)	3.27 (SD = 0.80)	3.34 (SD = 0.72)	2.43 (SD = 0.78)	2.56 (SD = 1.08)	3.34 (SD = 0.83)

^1^The MHAs Teen Counseling and Talkspace offer connections to licensed/trained counselors and therapists. Therapeutic persuasiveness and alliance may vary depending on the assigned counselor/therapist. For a reliable assessment multiple counselors and therapists would have been necessary, which was not possible within this study (→ NA)

### Associations between measures

Correlation between the overall MARS and ENLIGHT were high (*r* = .936, *p* < .001). No significant correlations between either MARS or ENLIGHT with user star rating were found (*p* > .050).

## Discussion

To the best of our knowledge, this is the first study that systematically evaluated the quality of available apps that were denoted to tackle symptoms of anxiety, depression and PTSD in youth. Although our systematic searches yielded in a high number of initial hits, only a very small fraction of apps (0.39%) fulfilled our eligibility criteria and were independently assessed with two established app rating scales. Overall, the results point to a mediocre quality of the 15 included apps, and most importantly, to a widespread absence of rigorous scientific evaluations of apps in RCT-studies.

Our quality ratings are in line with the results of previous systematic evaluations deploying the same rating scales on apps for various health conditions and application areas (e.g., depression, PTSD, pain, rheumatism, physical activity, weight management [16, 36, 38, 44–46, 52–54]), all indicating medium overall app qualities with the MARS and ENLIGHT scales. In our study, the categories with the highest ratings were aesthetics and functionality (M = 3.78; M = 3.81), followed by information quality (M = 3.42) and engagement (M = 3.34). Also corresponding to previous studies [16, 36], the most often deployed therapeutic background for health apps was CBT, with various intervention components applied such as psychoeducation, relaxation techniques, mindfulness, exposure or monitoring/tracking.

However, and most importantly, a common deficiency of MHA is the absence of empirical investigations on their efficacy [16, 36, 55], which represents a major limitation and confines further quality aspects in our view. Considering this lack of empirical support, it seems like a minor issue that youth, caregivers and mental health professionals are hardly able to disentangle the overabundance of available apps and to distinguish therapeutic relevant apps from those that are irrelevant for their health-related intentions [56]. Moreover, our results suggest that app users might not be able to sufficiently appraise the quality of available apps, given the discrepancy of expert and user ratings, which is consistent with findings from other studies [16]. Thus, it seems necessary that objective and scientifically-based expert ratings are publicly available, in order to enable patients to make informed and guided decisions about MHA in particular, and psychotherapeutic interventions in general, advocating evidence-based mental healthcare.

Next to several strengths (including the resort to established scientifically validated app rating scales, the focus on prevalent mental disorders and comprehensive scope on apps with divergent theoretical backgrounds), there are several limitations that ought to be considered when interpreting the findings of this current study. First, the search strategy embraced only available apps in the European *Google Play* and *Apple App* stores, and therefore the results might not be representative for other outlets and app stores. Second, the rapidly evolving dynamics of the app market [16, 36, 57] might have led to the omission of novel apps as well as updates of included apps, and that some of the apps might not be accessible anymore. Third, since the two rating scales were not designed for the specificities of child and adolescent psychotherapy (i.e., addressing relevant developmental issues), ratings of youth MHA should be interpreted with caution—especially in regard to the domains of functionality and information content. Therefore, it would be a significant scientific advancement, if specific MHA rating scales were available and psychometrically evaluated for mobile-based interventions for common mental disorders in youth. Fourth and foremost, quality ratings can never be sufficient to inform clinical practice and treatment approaches alone, since efficacy and effectiveness studies are the core and gold-standard of evidence-based mental healthcare.

Accordingly, the most important future direction and imperative necessity for the advancement of MHA, is their methodological sound and rigorous evaluation in randomized controlled trials (or novel alternative evaluation frameworks to MHA)—since only one app was subject to an efficacy study, with sobering non-significant findings [58]. Without comprehensive knowledge on their (non-)efficacy and intervention safety, these novel applications ought not to be part in routine mental healthcare. However, some stakeholders might compromise some established standards of evidence-based medicine and advocate the evaluation of MHA in routine clinical care – without prior information on their efficacy and harmlessness. This debatable procedure can be observed in the health sector in Germany, where clinicians and healthcare providers are currently entitled to prescribe apps, with the intention of the German health ministry and certifying agency (i.e., BfArM) to gain an evaluation of their effectiveness post-hoc (with app providers contributing these data themselves). As such, this development might bring several risks for individual patients (e.g., non-detected and unknown side effects, adverse events, or deterioration) and might contribute to an erosion of well-established standards of evidence-based medicine in general. This concern is also nourished by the massive commercialization of the app market and limited resources in healthcare globally, raising further methodological and ethical questions [59, 60]. Thus, the adherence to well-established standards of good clinical practices as well as scientific-based intervention evaluation should be urgently applied for MHA [61], similar as it is largely the case for internet-based interventions and conventional psychotherapies offered face-to-face. Recommendations for quality criteria and standards for MHA are available, and can complement efficacy-/effectiveness studies on MHA in RCTs [62, 63]. Furthermore, the development of MHA needs to get theoretically and empirically informed, since dismantling and additive design studies are largely pending in mobile-based interventions [18], and the resort to theoretically deduced and evidence-based intervention components is a critical step to build efficacious interventions that induce essential mechanisms of therapeutic change enabling improved outcomes [64].

Taken together, our study indicates that MHA for youth possess a rather small quality and, more importantly, are insufficiently evaluated concerning their efficacy, impeding widespread conclusions and recommendations for clinical practice and healthcare policies so far. Therefore, it is of utmost importance that patients and stakeholders are thoroughly and scientifically informed about this limited evidence base and the current deficiencies and restraints of MHA for youth. Information platforms offered by (non-profit) organizations, like www.mhad.science (last updated September 6, 2021) or https://onemindpsyberguide.org/ (last updated September 6, 2021), that gather and provide scientific knowledge on central aspects of digital health interventions (e.g., effectiveness, acceptability, quality, safety, or data security) in a balanced and unbiased way, are one important contribution for the evidence-based information and empowerment of patients seeking advice and help for their mental health resolutions.

## Conclusions

This study systematically evaluated the quality of MHA for anxiety, depression and PTSD in youth. The results revealed a moderate overall app quality and point to a significant lack of empirically-informed intervention development, as well as an absence of rigorous studies to investigate the efficacy of apps that are currently offered in major marketplaces. Hence, future high-quality research is urgently needed, to advance and evaluate MHA, all contributing to the augmentation and evidence-based utilization of digital health interventions. Furthermore, non-profit provisions of scientifically-based information on essential aspects of apps are urgently required to enable informed and balanced treatment choices for patients, caregivers and stakeholders in mental health.

## Supplementary Information

**Additional file 1.** Search terms.

## Data Availability

Data will be made available to researchers who provide a methodologically sound proposal, not already covered by other researchers. All requests should be directed to the corresponding author. Data requestors will need to sign a data access agreement. Provision of data is subject to data security regulations. Support depends on available resources.
