# Supplementary material for: Mobile-based interventions for common mental disorders in youth: a systematic evaluation of pediatric health apps
Source: Child Adolesc Psychiatry Ment Health. 2021 Sep 13;15:49. doi: 10.1186/s13034-021-00401-6 (PMC8438844; doi:10.1186/s13034-021-00401-6)
Supplement: Supplementary file 1 — Additional file 1. Search terms. [file 13034_2021_401_MOESM1_ESM.docx]

Appendix 1: Search terms.

The depression set included for example: *sleep problems*, *sleep trouble*, *depression*, *exhaust*, *sorrow*, *sadness*, *melancholy*, *despair*, *grief*, *rumination*, *depressive* *episode*, *depressive* *disorder*, *fatigue*, *tiredness*, *woe*, *unhappiness*; the anxiety terms were: *phobia*, *generalized* *anxiety* *disorder*, *separation* *anxiety*, *agora*, *anxiety*, *fear*, *nervousness*, *panic*, *panic* *attack*; and the terms *posttraumatic stress disorder*, *dissociation*, *traumatic* *stress*, *moral* *injury*, *flashback* and *irritability* were used to identify MHA for PTSD. Due the nature of the Google Play Store and Apple App Store API each terms was searched separately and truncation or logical operators could not be used.
